# The value of percutaneous ultrasound in predicting conversion from laparoscopic to open cholecystectomy due to acute cholecystitis

**DOI:** 10.1007/s00464-013-2787-9

**Published:** 2013-02-01

**Authors:** Grzegorz Ćwik, Tomasz Skoczylas, Justyna Wyroślak-Najs, Grzegorz Wallner

**Affiliations:** Second Department of General & Gastrointestinal Surgery & Surgical Oncology of the Alimentary Tract, Medical University of Lublin, 20-081 Lublin, ul. Staszica 16, Poland

**Keywords:** Acute cholecystitis, Laparoscopic cholecystectomy, Open cholecystectomy, Conversion, Percutaneous ultrasound

## Abstract

**Background:**

Laparoscopic cholecystectomy has become the treatment of choice for gallstone disease. Advantages of the laparoscopic approach include lower morbidity and mortality rates, reduced length of hospital stay, and earlier return to work. In acute cholecystitis, severe inflammation makes laparoscopic dissection technically more demanding, with a higher risk of related complications that require conversion to open cholecystectomy.

**Methods:**

We reviewed the records of 5,596 patients who underwent cholecystectomy between 1993 and 2011 in a single institution. A laparoscopic approach was undertaken in 4,105 patients (73.4 %). The ultrasound signs of acute cholecystitis were found in 542 patients (13.2 %) who underwent laparoscopic cholecystectomy. We analyzed the ultrasound presentations of acute cholecystitis in patients who required conversion to open cholecystectomy and compared them with the ultrasound signs of acute cholecystitis in patients who had a completed laparoscopic cholecystectomy.

**Results:**

A conversion to open cholecystectomy in patients with acute cholecystitis was necessary in 24 % (*n* = 130) of the patients compared to 3.4 % of the patients with uncomplicated gallstone disease. The most frequent ultrasound findings in patients requiring conversion were a pericholecystic exudate in 42 %, a difficult identification of anatomical structures due to local severe inflammation in 34 %, and gallbladder wall thickening of >5 mm in 31 %. Additionally, when the duration of symptoms exceeded 3 days, more than half of the patients required conversion to open cholecystectomy and the conversion rate was fivefold higher than for those with a shorter duration of acute cholecystitis.

**Conclusions:**

In patients with severe acute cholecystitis found on ultrasound, combined with gallbladder wall thickening to >5 mm, pericholecystic exudates or abscess adjacent to the gallbladder, difficulty identifying anatomical structures within Calot’s triangle, specifically when the duration of symptoms exceeds 3 days, cholecystectomy should be done as an open approach because of the high risk of conversion.

Laparoscopic cholecystectomy (LCH) has become the method of choice in the treatment of symptomatic uncomplicated gallstone disease. The advantages of the laparoscopic approach include a shorter hospital stay and recovery time, reduced postoperative pain, and better cosmetic results [1–6]. The role of LCH in the treatment of complicated gallstone disease, and particularly acute cholecystitis, is still controversial and has been a subject of vigorous debate [2, 7–9]. Severe inflammation makes laparoscopic dissection technically more demanding, with a higher wall perforation rate and spillage of the infected bile into the peritoneal cavity [2]. Infiltration of inflammation may disturb the anatomy of Calot’s triangle resulting in an increased risk of bile duct injury. Severe inflammation is also responsible for serious complications occurring in the postoperative course [10–12]. For several years after LCH had been introduced, some authors have suggested that acute cholecystitis is a contraindication for the laparoscopic approach [2, 6, 8]. However, with experience and increased skills in laparoscopic techniques, acute cholecystitis, excluding its gangrenous form, ceased to preclude LCH [3, 9, 13, 14].

Many publications pointed out that age over 60–65, previous upper abdominal surgery, clinical and ultrasound signs of severe acute cholecystitis at admission, and white blood count (WBC) >10.0 k/μL were significantly associated with conversion to open cholecystectomy (OCH) [1, 2, 5, 15]. Recently, an increasing number of reports have indicated that LCH is a safe method of treatment for acute cholecystitis, especially due to the improvement in surgical technique and instrumentation. The established limitations for use of LCH include the operating surgeon’s experience and the degree of inflammation, especially in seriously complicated cases [1, 2, 7, 13, 16]. Patients with acute cholecystitis are usually informed of the potential for conversion to open cholecystectomy when it is difficult to identify the main anatomic structures [5, 17, 18]. A conversion may prevent a severe complication such as injury to the biliary tree.

The aim of the study was to review comprehensively the indications and contraindications of the use of LCH in acute cholecystitis patients based on ultrasound presentation. Additional objectives of our analysis included identifying the most common causes of conversion from laparoscopic to open cholecystectomy and to define to what extent and in what cases it may be predicted via percutaneous ultrasound.

## Materials and methods

We retrospectively reviewed the records of 5,596 patients who underwent cholecystectomy for gallstone disease between 1993 and 2011 in a single tertiary-care institution (Second Department of General & Gastrointestinal Surgery & Surgical Oncology of the Alimentary Tract, Medical University of Lublin, Poland). The investigator (GĆ) who performed the preoperative ultrasound assessment was blinded to the intraoperative decision about conversion. A laparoscopic approach was undertaken in 4,105 patients (73.4 %). A decision about whether to initially use an open or a laparoscopic approach was made based on clinical presentation, ultrasound findings assessed in the department directly before the operation, and blood tests such as WBC and/or C-reactive protein.

On percutaneous ultrasound, special attention was paid to the thickness of the gallbladder wall, the echo pattern of the gallbladder lumen, the presence of fluid within the gallbladder wall or its closest surroundings, and fluid collections in the peritoneal cavity. The anatomic area covering the gallbladder neck, common bile duct (CBD), and neighboring vascular structures were subsequently identified. The intensity of infiltration of inflammation was assessed by the degree of obscuration of anatomic details within Calot’s triangle. Then, the width and contents of the CBD and the size and echogenic pattern of the pancreas were assessed.

Percutaneous ultrasound revealed signs of acute cholecystitis, such as wall thickening of >4 mm, stagnant or purulent gallbladder contents, and intramural or pericholecystic exudate, in 829 patients (14.8 % of 5,596 patients). LCH was undertaken in 542 patients (65.4 % of 829 patients) with acute cholecystitis. The remaining 287 patients (34.6 % of 829 patients) were primarily referred for OCH. The most frequent reasons for preclusion of LCH were signs of peritonitis, a suspicion of gangrenous cholecystitis or gallbladder wall perforation with an adjacent abscess, a rapid increase of bilirubin, a severe inflammatory infiltrate that hindered the interpretation of anatomic details on ultrasound, an emergency indication, or the patient’s refusal to undergo the laparoscopic approach.

An elevated bilirubin level was found on preliminary assessment in 187 patients (34.5 % of 542 patients) with acute cholecystitis who underwent LCH. In most cases it was caused by the inflammatory reaction in the region of the gallbladder neck and Calot’s triangle and was reduced after administration of antibiotics. In 61 patients (11.3 % of 542 patients) with acute cholecystitis who underwent LCH, ductal stones or dilatation of the CBD >10 mm was identified on ultrasound. In all these patients endoscopic sphincterotomy (ES) was performed before surgery, which was effective in 50 patients who were subsequently referred for LCH to be done the next day or 2 weeks after ES. The remaining 11 patients with ductal stones or dilated CBD qualified for OCH with bile duct exploration.

The initial classification of gallbladder inflammation as acute cholecystitis correlated with the histopathological evaluation of the removed specimen.

Additional ultrasound assessment was carried out if intense abdominal pain and fever persisted after the surgery and if laboratory tests revealed abnormalities, which usually occurred 2 or 3 days after the operation.

Follow-up was scheduled 30 days after surgery and 412 patients (76 %) showed up for the examination. Percutaneous ultrasound was used to evaluate the postcholecystectomy site, the width and shape of the CBD, and the echogenic pattern and size of the pancreas.

Statistical analysis was conducted using the STATISTICA software (StatSoft, Inc., Tulsa, OK). The differences between groups were compared using the χ^2^ test. A *P* value lower than 0.05 was regarded as significant.

## Results

We identified 542 patients with symptoms and signs of acute cholecystitis who were referred for LCH. All suffered from typical pain, intermittent to constant fever, increased WBC >10.0 k/μL, and at least one of the ultrasound features of acute cholecystitis mentioned above. Conversion from laparoscopic to open cholecystectomy was necessary in 130 patients (24 %). LCH was performed by a team led or supervised by an experienced laparoscopic surgeon (>100 LCH) who made the decision to convert. The conversion rate in patients who were undergoing LCH due to uncomplicated gallstone disease was 3.8 %. The reasons for conversion are listed in Table 1. The most common causes of conversion found in the initial phase of laparoscopy were the lack of confidence in identification of the cystic duct (36.2 %), massive inflammatory or postoperative adhesions (25.3 %), and flaccid gangrenous gallbladder (22.3 %). Common bile duct injuries occurred in three patients who underwent LCH for acute cholecystitis (0.5 %). The injuries were detected in all of them intraoperatively and required conversion to open access and were repaired with Roux-en-Y hepaticojejunostomy.

**Table 1:** The distribution of reasons for conversion from laparoscopic to open cholecystectomy in patients operated on for acute cholecystitis

Cause of conversion	*n*	%
Impossible identification of the cystic duct	47	36.2
Massive inflammatory or postoperative adhesions	33	25.3
Flaccid gangrenous gallbladder	29	22.3
Intensive bleeding	11	8.5
Necessity to explore the CBD	7	5.4
CBD injury	3	2.3
Total	130	

Comparison of the ultrasound presentation of the gallbladder and its surroundings for patients who required conversion to the open approach to the ultrasound findings for patients who underwent a completed LCH are given in Table 2. The most frequent findings on preoperative ultrasound in patients converted to OCH included obscured anatomy at the gallbladder neck, cystic duct and CBD linked to a local inflammatory infiltrate in 52 patients (40.0 %), pericholecystic exudate in 44 patients (33.8 %), and thickening of the gallbladder wall of more than 5 mm in 41 patients (31.5 %). Other ultrasound abnormalities occurred less frequently. In the patients who had a completed LCH, the ultrasound signs of acute cholecystitis were less intense. The most frequent findings included thickening of the gallbladder wall to 3–5 mm in 258 patients (62.6 %), a tense and enlarged gallbladder in 112 patients (27.2 %), pericholecystic exudate in 76 patients (18.4 %), and intramural exudate in 67 patients (16.3 %). Statistical analysis revealed significant differences between converted and completed LCH patients. The thickening of the gallbladder wall to more than 5 mm (31.5 vs. 8.8 %, *P* < 0.000001), pericholecystic exudate (33.8 vs. 18.4 %, *P* = 0.002), abscess adjacent to the gallbladder (17.7 vs. 6.6 %, *P* = 0.0001), intensive gallbladder wall deformation (21.5 vs. 9.2 %, *P* = 0.0002), and difficulty identifying anatomical structures (40.0 vs. 23.3 %, *P* = 0.003) more frequently predicted conversion to OCH. On the other hand, the thickening of the gallbladder wall to 3–5 mm more frequently predicted successful completion of LCH (62.6 vs. 13.8 %, *P* < 0.000001). The analysis of the ultrasound findings for patients with acute cholecystitis who were undergoing LCH and required conversion did not reveal significant differences between those who were operated on in the early period (1993–2001) and those in the late period (2002–2011) (Table 2).

**Table 2:** Ultrasound presentation of the gallbladder in patients with acute cholecystitis who underwent laparoscopic cholecystectomy and required conversion to the open approach

Ultrasound finding	CONV		LCH			CONV I		CONV II	
	*n*	%	*n*	%	*P*	*n*	%	*n*	%
GB wall thickening 3–5 mm	18	13.8	258	62.6	<0.000001	10	14.5	8	13.1
GB wall thickening >5 mm	41	31.5	36	8.8	<0.000001	22	31.9	19	31.1
Intramural exudate	24	18.5	67	16.3	0.56	16	23.2	8	13.1
Pericholecystic exudate	44	33.8	76	18.4	0.002	25	36.2	19	31.1
Suspected GB wall gangrene	21	16.1	42	10.2	0.06	10	14.5	11	18.0
Pericholecystic abscess	23	17.7	27	6.6	0.0001	13	18.8	10	16.4
Intensive GB wall deformation	28	21.5	38	9.2	0.0002	15	21.7	13	21.3
Difficult identification of anatomical structures	52	40.0	96	23.3	0.003	26	37.7	26	42.6
Tense, enlarged GB	31	23.8	112	27.2	0.45	15	21.7	16	26.2
Total number of patients	130		412			69		61	

The differences between groups were calculated using χ^2^ test; *P* is the level of significance

*LCH* patients who had a completed laparoscopic cholecystectomy, *CONV* patients who started with a laparoscopic cholecystectomy and then required conversion to open cholecystectomy, *CONV I* patients who started with laparoscopic cholecystectomy and then required conversion to open cholecystectomy between 1993 and 2002, *CONV* *II* patients who started with laparoscopic cholecystectomy and then required conversion to open cholecystectomy between 2003 and 2011, *GB* gallbladder

In patients who required conversion, ultrasound findings of acute cholecystitis usually were combined with two or three other components such as concomitant thickening of the gallbladder wall to more than 5 mm, obscured anatomy of Calot’s triangle, and pericholecystic exudate or abscess. The number of concomitant ultrasound findings had considerable influence on the risk of conversion, especially in patients with three simultaneous findings (Table 3). A combination of at least two of these findings with acute cholecystitis resulted in a conversion rate >70 %. Under these circumstances, slow dissection and difficulty in identifying anatomical structures frequently and remarkably limited even the experienced laparoscopist.

**Table 3:** Number of ultrasound findings identified simultaneously with acute cholecystitis in patients who underwent conversion from laparoscopic to open cholecystectomy

No. of ultrasound findings	*n*	%
1	35	26.9
2	38	29.2
3	57	43.9
Total No. of patients	130	

**Fig. 1:**
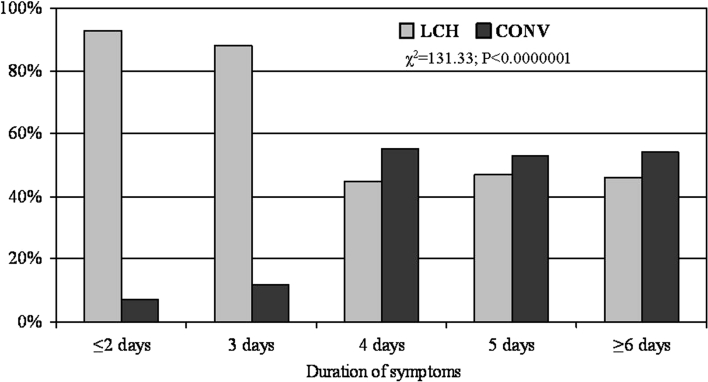
Time from the onset of symptoms of acute cholecystitis to surgery in patients who underwent laparoscopic cholecystectomy and required conversion. *LCH* patients who had a completed laparoscopic cholecystectomy, *CONV* patients who started with laparoscopic cholecystectomy and then required conversion to open cholecystectomy, P is the level of significance, and the differences between groups were calculated using χ^2^ test

Additionally, the conversion rate significantly correlated with duration of symptoms of acute cholecystitis (Fig. 1). The mean time from the onset of symptoms to surgery for patients who underwent LCH and required conversion was 1.26 days longer than for patients who underwent completed LCH (Table 4). When the duration of symptoms was 4 or more days, more than half of the patients required conversion to open cholecystectomy and the conversion rate was fivefold higher than in patients with a shorter duration of symptoms (Fig. 1; Table 4).

**Table 4:** Time from the onset of symptoms of acute cholecystitis to surgery in patients who underwent laparoscopic cholecystectomy and required conversion

Duration of symptoms	LCH		CONV		
	*n*	%	*n*	%	*P*
≤2 days	168	92.8	13	7.2	<0.0000001
3 days	164	88.2	22	11.8	
4 days	33	44.6	41	55.4	
5 days	30	46.9	34	53.1	
≥6 days	17	45.9	20	54.1	
Total no. of patients	412		130		
Mean duration of symptoms (days)	2.94		4.20		

*P* is the level of significance, the differences between groups were calculated using χ^2^ test (χ^2^=131.33)

*LCH* patients who had a completed laparoscopic cholecystectomy, *CONV* patients who started with laparoscopic cholecystectomy and then required conversion to open cholecystectomy

On the follow-up ultrasound performed 30 days after LCH, a CBD dilated to >10 mm was found in 25 of 412 patients (6.0 %). Only 9 (2.2 %) symptomatic patients with confirmed ductal stones required ES and stone extraction.

## Discussion

In the last two decades, LCH has become the standard treatment of symptomatic gallstone disease [2, 4, 5, 19]. LCH is a widely recommended method for both complicated and uncomplicated disease [3, 8, 13, 16]. Conversion to OCH is occasionally necessary to avoid injury to the CBD and to clarify obscured anatomy. The conversion rate in uncomplicated gallstone disease is low, ranging between 1 and 6 %; in our series it reached 3.8 % [5, 11, 13–15]. Slim women under 65, with a short duration of the disease and an uncomplicated presentation on ultrasound, are at the lowest risk of conversion [5, 11].

In patients with acute cholecystitis, the conversion rate is higher, i.e., 22 % [2, 7, 11, 20, 21], and in some reports it is as high as 35 % [4, 9, 13, 18, 22]. In our series the conversion rate in acute cholecystitis patients was 24 %. Factors that increase the risk of conversion include the duration of acute symptoms, great damage to the gallbladder wall, inflammatory infiltration of the components of Calot’s triangle, adhesions in the upper part of the abdominal cavity, and intraoperative complications. It has been established that 72–96 h from admission to the hospital is the optimal time to perform cholecystectomy in acute cholecystitis, since thereafter the risk of complications increases significantly [4, 6, 14]. If an elevated WBC does not decrease and the clinical presentation is not alleviated within 4 days while on antibiotic therapy, the patient should be referred for OCH [17]. Few studies have focused on the proper timing of surgical intervention for acute cholecystitis, but, in general, 4 days of complaints has been proposed as the cutoff point after which the conversion rate rises significantly [4, 6, 14, 23, 24]. Our data showed that if the symptoms lasted 4 or more days, more than half of the patients required conversion to open cholecystectomy and the conversion rate was fivefold higher than in patients with a shorter duration of symptoms (Table 4; Fig. 1).

Previous upper abdominal surgery increases the risk for conversion to OCH, up to as much as 25 % [5, 19, 25]. Peritoneal adhesions make access to the gallbladder difficult. Dense adhesions surrounding the gallbladder and adhesions between the CBD, cystic duct, and cystic artery (Calot’s triangle) clearly obscure the field of view during surgery. These conditions may significantly prolong laparoscopic dissection and may result in bleeding or gallbladder rupture [2, 26]. In patients suspected of having dense adhesions, the open technique should be the initial treatment of choice [5, 6, 16, 25].

The extent of the weakening of the gallbladder wall is another cause of an increased risk of conversion [2, 13, 26, 27]. A gangrenous gallbladder may result in a disruption of wall continuity, flaccidity, and tendency to laceration during retraction and removal. Inflammation of the gallbladder greatly increases the risk of wall damage up to 34 %, which in turn may result in a higher rate of conversion, reaching 23 % [2]. Some authors regard a gangrenous gallbladder as direct indication for choosing the open approach due to a high rate of conversion and postoperative complications [13, 14]. This is exemplified by the increased conversion rate associated with the different degrees of inflammation: 40 % in gangrenous cholecystitis [4], 71 % in phlegmonous cholecystitis [17], and 12 % in empyema of the gallbladder [15].

Thickening of the gallbladder wall to >5 mm that is related to ongoing acute inflammation and inflammatory infiltration of the neck and Calot’s triangle are other important causes of conversion [3–5, 10, 23]. Severe inflammation in this crucial anatomic area make some surgeons perform such unconventional procedures as laparoscopic subtotal cholecystectomy [10] or percutaneous cholecystostomy [12] because of difficulty in identifying the local anatomy and the risk of injury to important structures such as the CBD and the hepatic artery. For surgeons with low experience, if a bile duct injury does occur, it is usually during the first 25 cholecystectomies [9]. It occurs less frequently for experienced laparoscopists who are able to evaluate the risk properly and make the decision early to convert [2, 5, 23]. The level of experience of the surgeon has a considerable impact on conversion rate, with longer operation times and more intraoperative complications for junior residents [19, 28]. Increased experience in preoperative and intraoperative assessment results in a change of distribution between LCH and OCH [2, 4, 18] and the majority of conversions occur in the first months and years of the learning curve [4, 9, 16, 18, 29]. That was the case in our department, where LCH was preferred in only 62–73 % of symptomatic uncomplicated gallstone disease cases during the first 4 years compared with 95 % of the cases in the last 10 years. Moreover, LCH is preferred more frequently for patients with acute cholecystitis, where the only contraindication is severe clinical signs accompanied by multiple simultaneous findings of severe inflammation on ultrasound.

One of the most severe complications of cholecystectomy is injury to the CBD. For OCH, the prevalence of bile duct injury has been estimated to be 0.1–0.2 %, but for LCH, the rate has been reported as high as 0.5 % [5, 9, 30, 31]. The most common causes of CBD injury are the failure to recognize the anatomy of Calot’s triangle, inadequate experience of the surgeon, and local anatomical risk factors. In our series of patients with acute cholecystitis, we identified three cases of serious CBD injury during LCH (0.55 %). All were detected intraoperatively and required conversion to open access and were repaired with Roux-en-Y hepaticojejunostomy.

Conversion to OCH has been associated with a longer operative time, the use of more anesthetics, increased overall morbidity, a higher rate of infective complications, longer recovery time, longer hospital stay, higher cost, and greater patient dissatisfaction [5, 19, 29, 32–34]. Thus, the ability to predict preoperatively the technical difficulties that may occur during surgery in order to choose between the laparoscopic or the open approach is desirable.

Concomitant choledocholithiasis, usually found in 10–12 % of the cases, is not a direct contraindication for LCH as long as ductal calculi are removed endoscopically before or sometimes after the operation, or during LCH by transcystic or direct laparoscopic CBD exploration [9, 26, 27, 29, 35]. The principal issue is to establish indications for ES. The primary diagnostic imaging method used to check for indications to use ES and CBD exploration is percutaneous ultrasound, although its limitations in the evaluation of the bile ducts narrow its sensitivity to 68–94 % [32]. Moreover, serum testing for bilirubin level, alkaline phosphatase, liver transaminases, and lipase should be assessed initially and monitored during the course of the disease [14, 19, 27, 32, 35]. In most cases, a raised bilirubin level in acute cholecystitis results from inflammatory infiltration of Calot’s triangle and decreases after the implementation of antibiotic therapy [4, 17, 33]. An elevated bilirubin level may also be associated with Mirizzi syndrome, which in turn may increase the probability of conversion [19, 36].

A thickening and deformation of the gallbladder wall and signs mimicking gross inflammation in patients over 60 may result from the gallbladder cancer, which occurs in 3–10 % of gallbladder disease cases [20, 22]. LCH is not recommended in cancer patients as it generally does not provide a potential for cure and may contribute to peritoneal dissemination and port site metastasis [20]. If cancer is found during LCH, conversion to open and more radical surgery, including removal of the port sites, should be performed in all resectable cases [22].

Acute cholecystitis does not preclude LCH. However, there are certain constraints in the application of this technique, particularly in patients with a gross inflammatory reaction [10, 13, 17]. Is it possible to establish the contraindications for LCH? We can try to answer this difficult question by identifying the causes of conversion. The compiled statistical analysis failed to define predictors unequivocally. A considerable thickening of the gallbladder wall, impeded orientation in cirrhotic changes, gallstone disease in men over 65, and palpable gallbladder mass on physical examination were found to be the most frequent causes of conversion [1, 15, 21, 24, 34]. Additional significant reasons for conversion to OCH include intraperitoneal adhesions, obscured anatomy, obesity, old age, male gender, persistent fever, and ultrasound findings of acute cholecystitis [5, 11, 15, 17–19, 31, 33]. An elevated level of alkaline phosphatase >200 IU, amylase ≥150 U/L, lipase ≥128 U/L, WBC > 14.0 k/μL, APACHE II score >10 points, and raised LDH level significantly also increase the risk of conversion [11, 19, 33]. In multivariate analysis, a body mass index (BMI) >25–30 kg/m^2^ was identified as an important predictor of conversion [1, 15, 19]. Additionally, a delay of surgery of more than 72 h from the time of admission with concomitant acute symptoms of inflammation was frequently associated with conversion [19, 23]. All of the above-mentioned scoring systems and predictive factors have demonstrated inconsistent value, and none has been widely incorporated into surgical practice [19].

**Fig. 2:**
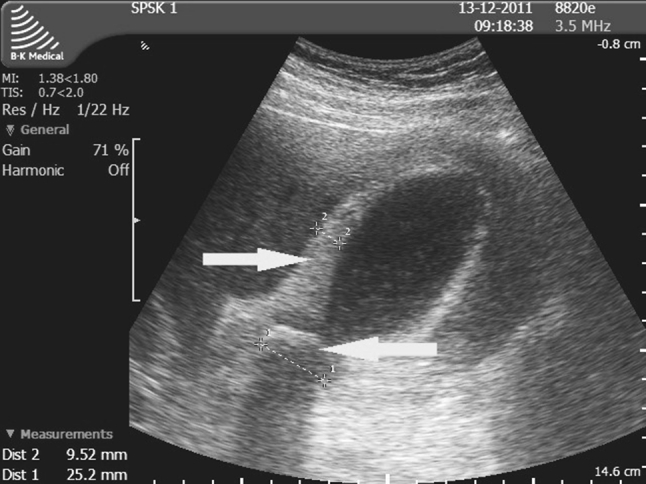
Ultrasound findings in acute cholecystitis that could affect the outcome of surgery: severe thickening of the gallbladder wall (8–9 mm), a large stone obstructing the gallbladder neck, and deformation of Calot’s triangle

**Fig. 3:**
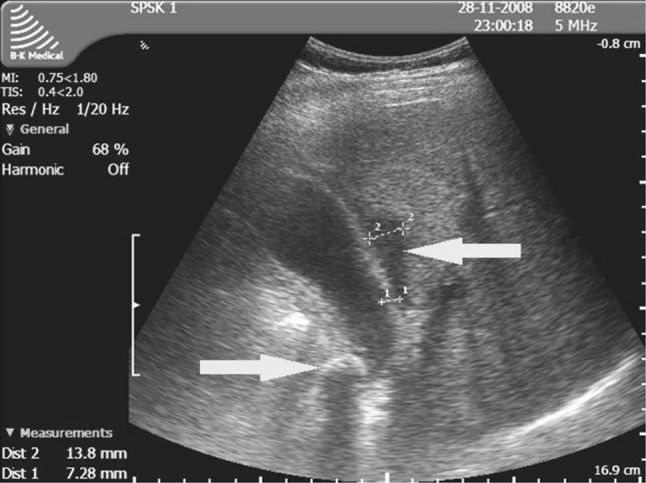
Ultrasound findings in acute cholecystitis that could affect the outcome of surgery: the gallbladder obstructed by a large stone, inflammation of the gallbladder wall, and pericholecystic exudate separating the liver bed

**Fig. 4:**
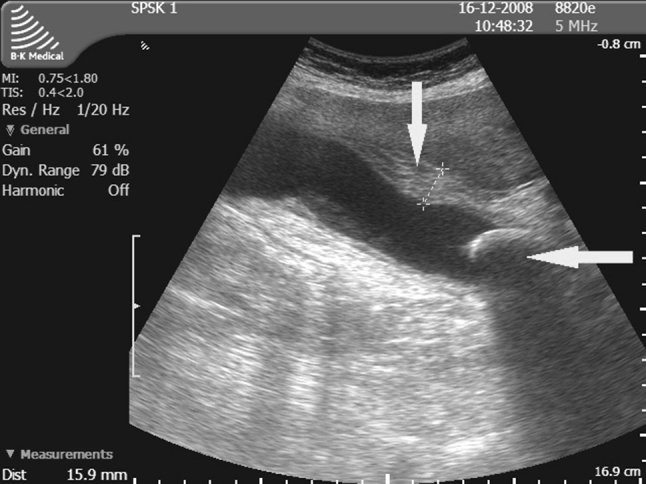
Ultrasound findings in acute cholecystitis that could affect the outcome of surgery: the gallbladder with a severely thickened wall, obstructed by a stone, with pericholecystic exudate; large deformation of Calot’s triangle makes identification of anatomical structures impossible

**Fig. 5:**
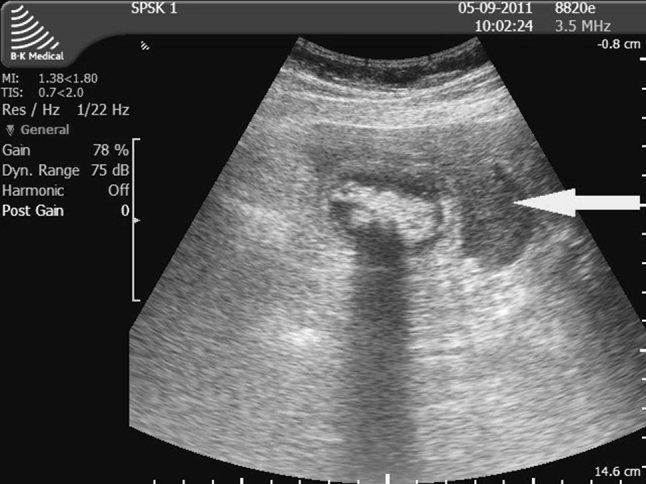
Ultrasound findings in acute cholecystitis that could affect the outcome of surgery: a severe inflammation and thickening of the gallbladder wall; on the liver side a fluid collection (pericholecystic abscess) is visible

Preoperative diagnostic procedures should support the decision for the appropriate surgical approach. Percutaneous ultrasound is a simple diagnostic technique that provides essential information on inflammatory complications that could affect the outcome of surgery. The most commonly discussed ultrasound findings include thickening of the gallbladder wall to ≥4 mm (Fig. 2) [5, 6, 18, 21, 29], pericholecystic exudate (Fig. 3) [7, 15, 19, 37, 38], edematous gallbladder wall (intramural exudate) (Fig. 4) [1, 15], Murphy’s sign on ultrasound [5, 15, 26, 36, 37], a distended gallbladder obstructed by stones [1, 6, 18, 29, 36], and pericholecystic abscess (Fig. 5) [1]. Ultrasound signs of inflammation result in a 7–8.5-fold higher rate of conversion to OCH [3, 15, 19, 33]. Other ultrasound findings that can predict technical difficulties during LCH include excessive gallbladder volume, impacted stones in the gallbladder neck, ductal stones, size of the largest gallstone, color or power Doppler signs within the gallbladder wall or in the adjacent liver, and pattern of gallbladder wall thickening [6, 18, 19, 25, 29, 34, 37].

Our large series identified ultrasound presentations of acute cholecystitis, such as gallbladder wall thickening to more than 5 mm, pericholecystic exudate, abscess adjacent to the gallbladder, intense gallbladder wall deformation, and difficulty in identifying anatomical structures, as significant predictive factors of conversion to OCH. On the other hand, gallbladder wall thickening to 3–5 mm more frequently predicted successful completion of LCH. Moreover, ultrasound presentation of a combination of at least two of the following signs: thickening of the gallbladder wall to more than 5 mm, blurred anatomy of Calot’s triangle, and pericholecystic exudate or abscess, resulted in a conversion rate exceeding 70 %. Therefore, ultrasound assessment assists the surgeon in preoperative or intraoperative decision making. In patients with severe inflammation found on ultrasound combined with gallbladder wall thickening to more than 5 mm, pericholecystic exudate or abscess adjacent to the gallbladder, and difficulty in identifying anatomical structures within Calot’s triangle, specifically when the symptoms have lasted more than 3 days, open cholecystectomy should be used because the risk of conversion from LCH is high, even for the experienced laparoscopic surgeon. This approach may save operating time and reduce overall cost by not having to convert from LCH [6, 37, 38].
